# Activation of the β-carbonic anhydrase from the protozoan pathogen *Trichomonas vaginalis* with amines and amino acids

**DOI:** 10.1080/14756366.2021.1897802

**Published:** 2021-03-10

**Authors:** Andrea Angeli, Linda J. Urbański, Vesa P. Hytönen, Seppo Parkkila, Claudiu T. Supuran

**Affiliations:** a Neurofarba Department, Sezione di Chimica Farmaceutica e Nutraceutica, Università degli Studi di Firenze, Firenze, Italy; b Faculty of Medicine and Health Technology, Tampere University, Tampere, Finland; c Fimlab Ltd, Tampere, Finland

**Keywords:** Amine, amino acid, carbonic anhydrase, activator, *Trichomonas vaginalis*

## Abstract

We report the first activation study of the β-class carbonic anhydrase (CA, EC 4.2.1.1) encoded in the genome of the protozoan pathogen *Trichomonas vaginalis*, TvaCA1. Among 24 amino acid and amine activators investigated, derivatives incorporating a second carboxylic moiety, such as L-Asp, L- and D-Glu, were devoid of activating effects up to concentrations of 50 µM within the assay system, whereas the corresponding compounds with a CONH_2_ moiety, i.e. L-Gln and L-Asn showed modest activating effects, with activation constants in the range of 26.9 − 32.5 µM. Moderate activation was observed with L- and D-DOPA, histamine, dopamine, serotonin, (2-Aminoethyl)pyridine/piperazine and morpholine (K_A_‘s ranging between 8.3 and 14.5 µM), while the best activators were L-and D-Trp, L-and D-Tyr and 4-amino-Phe, which showed K_A_‘s ranging between 3.0 and 5.1 µM. Understanding in detail the activation mechanism of β-CAs may be relevant for the design of enzyme activity modulators with potential clinical significance.

## 1. Introduction

Diseases provoked by protozoan pathogens are widespread and few effective agents for their treatment are available^1–4^. Furthermore, most of the drugs in clinical use are either dating back to the 50 s or the 60 s and are thus rather toxic and poorly effective, and/or extensive drug resistance has been developed to most of them in many places all over the world, creating thus pressure on the healthcare systems and a large number of casualties^1–4^. This is particularly the case with malaria, caused by protozoans belonging to the genus *Plasmodium*, with five different species infecting humans, *P. falciparum*, *P. vivax*, *P. ovale*, *P. malariae*, and the zoonotic *P. knowlesi*^1^, but also with other pathogens, such as *Trypanosoma cruzi* and *T. brucei*, provoking Chagas disease and African trypanosomiasis, respectively^2^, various species of *Leishmania*, which provoke leishmaniasis^3^, or *Trichomonas vaginalis* which is one of the most common such pathogens, to mention just few of them. Due to the lack of new drugs and the poor response to those available, alternative drug targets for fighting such diseases are constantly being looked for and proposed^1^^,^^5^^,^^6^. Interesting novel strategies for drug development involve inhibition of carbonic anhydrases (CAs, EC 4.2.1.1) from pathogenic protozoans^1^^,^^2^^,^^5^^,^^6^. Indeed, it has been reported that strong anti-protozoan effect especially against *T. cruzi* as well as several *Leishmania* species can be achieved by inhibiting CAs with potent and in some cases specific CA inhibitors (CAIs)^1^^,^^2^^,^^5^^,^^6^. On the other hand, CA activators (CAAs) of such protozoan enzymes have been much less investigated, and in fact only two such reports are available in the literature. They include the activation study of the β-CA from *L. donovani chagasi* and *Entamoeba histolytica*, which were in fact reported by our groups^7^. CAAs started to be considered only recently for their potential clinical applications^8^, and at least activation of the human CA (hCA) isoforms was demonstrated to be of interest for the modulation of emotional memory as well as the extinction of contextual fear memory, which opens relevant pharmacological applications for this class of compounds^8^.

*Trichomonas vaginalis,* the anaerobic protozoan responsible for the most frequent non-viral sexually transmitted disease in humans^9^, has recently been investigated for the presence of CAs. Indeed, at least two such enzymes belonging to the β-CA class are present in its genome, TvaCA1^9^^,^^10^ and TvaCA2. Both the structure and catalytic properties of TvaCA1 have been characterised by X-ray crystallography and kinetic techniques, which showed it to be an efficient catalyst for the interconversion between CO_2_ and bicarbonate in the reaction which also generates protons. This reaction is probably an essential part of the molecular machinery involved in the pH regulation and metabolism of the parasite^9^. Furthermore, anion and sulphonamide inhibition studies of this enzyme were reported^9^^,^^10^. Since humans do not have β-CAs in their genomes, but only α-class CA enzymes^11–13^, some of which are well-known drug targets, modulation of TvaCA1 activity (and probably also the other isoform) might represent an interesting option for finding anti-protozoan agents with a novel mechanism of action^1^. Although activation of pathogenic CAs may be detrimental for the host organism, this phenomenon should also be investigated in detail. Importantly, many CAAs belong to the amine and amino acid classes and several of these compounds are endogenous and present in high concentrations in various tissues/cells, and thus may participate in the modulation of infection and virulence by the pathogen^14^. Here we report the first activation study of the β-CA from *T. vaginalis* TvaCA1, with a series of amines and amino acids, many of which are naturally occurring compounds.

## 2. Materials and methods

### 2.1. Chemistry

Compounds **1–24** are commercially available, highest purity reagents, from Sigma-Aldrich (Milan, Italy).

### 2.2. Enzymology

TvaCA1 was a recombinant enzyme obtained in-house as described earlier^9^. Briefly, the *TvaCA1* gene was identified from the Universal Protein Resource Database Uniprot (Protein entry: A2ENQ8). Gene synthesis and subcloning were performed by GeneArt (Thermo Fisher Scientific, Germany). TvaCA1 was expressed recombinantly in *E. coli* (OneShot^®^ BL21 Star™ (DE3) Chemically Competent Cells, #C601003, Thermo Fisher Scientific, Finland). The recombinant protein was purified using Ni^2+^-NTA Agarose affinity chromatography resin (Macherey-Nagel GmbH Co., Germany). The 6xHis-tag was removed by thrombin (#RECOMT, Sigma-Aldrich, Finland) according to Thrombin CleanClive™ kit manual (Sigma-Aldrich, Finland), and the tag was separated from the core protein by Ni^2+^-NTA affinity chromatography.

### 2.3. Ca activity/activation measurements

An Sx.18Mv-R Applied Photophysics (Oxford, UK) stopped-flow instrument has been used to assay the catalytic activity of various CA isozymes for CO_2_ hydration reaction^15^. Phenol red (at a concentration of 0.2 mM) was used as an indicator, working at the absorbance maximum of 557 nm, with 10 mM TRIS (pH 8.3, for β-CAs)^7^ as buffers, 0.1 M NaClO_4_ (for maintaining constant ionic strength), following the CA-catalyzed CO_2_ hydration reaction for a period of 10 s at 25 °C. The CO_2_ concentrations ranged from 1.7 to 17 mM for the determination of the kinetic parameters and inhibition constants. For each activator at least six traces of the initial 5–10% of the reaction have been used for determining the initial velocity. The uncatalyzed rates were determined in the same manner and subtracted from the total observed rates. Stock solutions of activators (at 0.1 mM) were prepared in distilled-deionized water and dilutions down to 1 nM were made thereafter with the assay buffer. Enzyme (in the concentration range of 8–15 nM) and activator solutions were pre-incubated together for 15 min prior to assay, in order to allow for the formation of the enzyme–activator complexes. The activation constant (K_A_), defined similarly with the inhibition constant K_I_, can be obtained by considering the classical Michaelis–Menten equation (Equation (1), which has been fitted by non-linear least squares by using PRISM 3:

(1) $$v\;=v_{\max}/\{1+(K_{M}/[S])(1+{\left[A\right]}_{f}/K_{A})\}$$

where [A]_f_ is the free concentration of activator.

Working at substrate concentrations considerably lower than K_M_ ([S] ≪K_M_), and considering that [A]_f_ can be represented in the form of the total concentration of the enzyme ([E]_t_) and activator ([A]_t_), the obtained competitive steady-state equation for determining the activation constant is given by Equation (2):

(2) $$v=v_{0}.K_{A}/\{K_{A}+({\left[A\right]}_{t}-0.5\{({\left[A\right]}_{t}+{\left[E\right]}_{t}+K_{A})-{\left({\left[A\right]}_{t}+{\left[E\right]}_{t}+K_{A}\right)}^{2}-{4{\left[A\right]}_{t}.{\left[E\right]}_{t})}^{1/2}\}\}$$

where v_0_ represents the initial velocity of the enzyme-catalyzed reaction in the absence of activator^16–19^. This type of approach to measure enzyme-ligand interactions is in excellent agreement with recent results from native mass spectrometry measurements^20^.

## 3. Results and discussion

TvaCA1 is a β-CA that has an open active site^21^, meaning that the water molecule/zinc hydroxide acting as nucleophile in the catalytic cycle is coordinated to the metal ion.

As all β-CAs, TvaCA1 is a homodimer, possessing two long channel-like active sites in its molecule, as determined recently by X-ray crystallographic techniques^9^. Thus, this enzyme is rather different from the α-CAs present in the human host, which are generally monomeric enzymes with the zinc ion coordinated by three His residues and a water molecule, therefore possessing a rather ample active site where inhibitors and activators may bind^11–13^. On the other hand, in TvaCA1 as in many β-CAs, the zinc ion is coordinated by two Cys residues, one His and one water molecule/hydroxide ion^9^^,^^21–23^. The rate-determining step for many CAs is the generation of the zinc hydroxide, nucleophilic species of the enzyme^11–13^. In α-CAs, this proton transfer reaction from the water molecule coordinated to the zinc to the reaction medium, is assisted by a His residue placed in the middle of the active site cleft, i.e. His64 in most hCA isoforms^11–13^. For β-class enzymes, the nature and position of the proton shuttling moiety are less well understood, being more complex than in the α-CAs. For example, recent X-ray crystallographic and mutagenesis studies^22^ allowed us to propose Asp309 as the proton shuttling residue for the β-CA PtLCIB3 of the diatom *Phaeodactylum tricornutum*, which differs substantially from the mechanism in α-CAs, for which a His residue, as mentioned above, has this role. It should be however mentioned that in another β-CA, the enzyme from *Pisum sativum*, the proton shuttle seems to be a Tyr residue^23^. Thus, it is obvious that both the catalytic as well as the activation mechanisms of β-CAs might be more complex than for the well studied α-class enzymes, and investigating β-CAs activators might be relevant also from this viewpoint.

**Figure 1.**
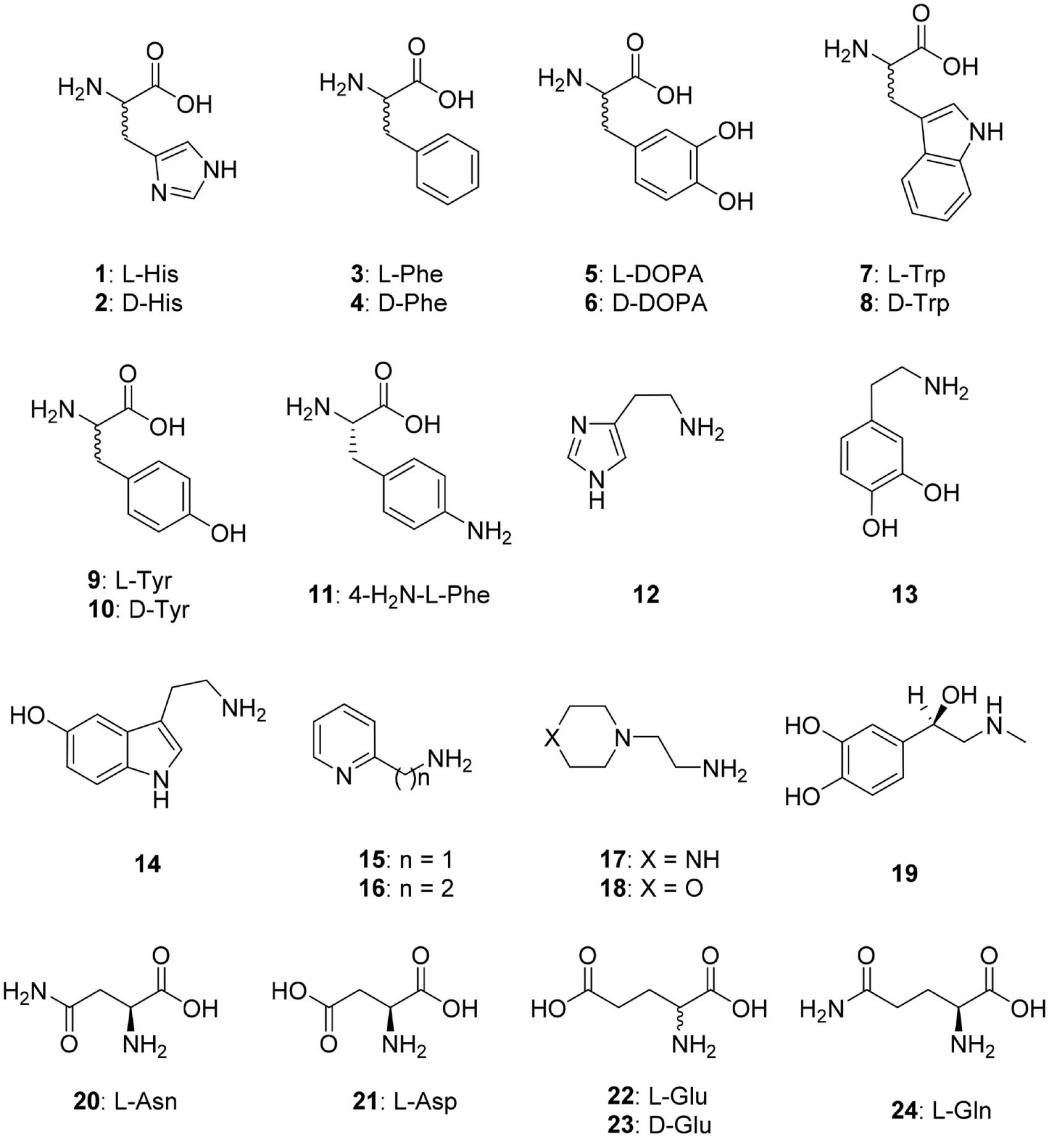
Amino acids and amines **1–24** investigated as CAAs against TvaCA1. Some β-CAs have a so-called closed active site (at pH < 8.3), in which the fourth zinc ligand is an aspartate residue, and thus these enzymes are devoid of CO_2_ hydrase activity^21–23^. However, at pH values > 8.3, the aspartate is involved in a hydrogen bond with an adjacent Arg residue, and an incoming water molecule/hydroxide ion replaces the aspartate as a zinc ligand, providing an open active site and thus a catalytically active enzyme^21–23^.

A panel of amino acid and amine derivatives of types **1–24** (Figure 1) were included in this study for investigating their activating properties against TvaCA1. These compounds were shown previously to act as CAAs against a range of CAs belonging to all known genetic CA families, including some α- and β-class enzymes^8^^,^^14^^,^^24^.

We have first investigated whether the activators of TvaCA1 interfere with the binding of the substrate CO_2_ to the enzyme or whether they only contribute to the proton transfer processes, as for other CAs investigated so far for their activation. As seen from data of Table 1, L-Trp, at 10 µM, efficiently activates TvaCA1 (as well as other α- and β-CAs, such as hCA I and II or the β-CA from *Escherichia coli*, EcoCAβ) inducing a 6.2-times increase in the k_cat_ of TvaCA1 but having no influence on K_M_, thus proving that the activator takes part in the proton transfer process within the enzyme-activator complex formed when the amino acid activator binds within the enzyme active site. However, this process appears not to interfere with the binding of CO_2_, since the value of K_M_ is not changed (Table 1).

**Table 1.** Activation of hCA isozymes I, II, EcoCAβ and TvaCA1 with L-Trp, measured at 25 °C^15^.

Isozyme	class	k_cat_* (s^−1^)	K_M_* (mM)	(k_cat_)_L-Trp_** (s^−1^)	K_A_*** (µM) L-Trp
hCA I^a^	α	2.0 × 10^5^	4.0	3.4 × 10^5^	44.0
hCA II^a^	α	1.4 × 10^6^	9.3	4.9 × 10^6^	27.0
EcoCAβ^b^	β	5.3 × 10^5^	12.9	1.8 × 10^6^	18.3
TvaCA1^c^	β	4.9 × 10^5^	6.1	3.0 × 10^6^	5.1

*Observed catalytic rate without an activator. K_M_ values in the presence and absence of activators were the same for the various CAs (data not shown).

**Observed catalytic rate in the presence of 10 µM activator.

***The activation constant (K_A_) for each enzyme was obtained by fitting the observed catalytic enhancements as a function of the activator concentration^15^.

^a^Human recombinant isozymes, from ref.^11^. ^b^Bacterial recombinant enzyme, from ref.^14^^b^. ^c^This work, protozoan enzyme. All values are mean from at least three determinations by the stopped-flow, CO_2_ hydrase method^15^. Errors were in the range of 5–10% of the reported values (data not shown).

We have thereafter investigated the amino acids and amines **1–24** for their effects on the TvaCA1 activation, comparing this data with those for hCA I, II and EcoCAβ (Table 2). The following structure-activity relationship for the activation of the protozoan enzyme was possible to draw from the data of Table 2:

**Table 2.** Activation constants of hCA I, hCA II and the bacterial enzyme EcoCAβ (*E. coli*) and the protozoan TvaCA1 (*T. vaginalis*) with amino acids and amines **1–24**, by a stopped-flow CO_2_ hydrase assay^15^.

		K_A_ (µM)*			
No.	Compound	hCA I^a^	hCA II^a^	EcoCAβ^b^	TvaCA1^c^
**1**	L-His	0.03	10.9	36.0	20.1
**2**	D-His	0.09	43	23.7	24.5
**3**	L-Phe	0.07	0.013	12.0	23.6
**4**	D-Phe	86	0.035	15.4	16.3
**5**	L-DOPA	3.1	11.4	10.7	12.1
**6**	D-DOPA	4.9	7.8	3.14	11.0
**7**	L-Trp	44	27	18.3	5.1
**8**	D-Trp	41	12	11.5	3.6
**9**	L-Tyr	0.02	0.011	9.86	4.9
**10**	D-Tyr	0.04	0.013	17.9	3.0
**11**	4-H_2_N-L-Phe	0.24	0.15	7.34	3.5
**12**	Histamine	2.1	125	18.5	8.4
**13**	Dopamine	13.5	9.2	11.3	12.6
**14**	Serotonin	45	50	2.76	9.1
**15**	2-Pyridyl-methylamine	26	34	48.7	9.5
**16**	2-(2-Aminoethyl)pyridine	13	15	17.2	12.0
**17**	1-(2-Aminoethyl)-piperazine	7.4	2.3	14.1	11.8
**18**	4-(2-Aminoethyl)-morpholine 0.14	0.19	17.4	14.5	
**19**	L-Adrenaline	0.09	96.0	9.15	8.3
**20**	L-Asn	11.3	>100	49.5	32.5
**21**	L-Asp	5.20	>100	18.9	>50
**22**	L-Glu	6.43	>100	18.0	>50
**23**	D-Glu	10.7	>100	11.4	>50
**24**	L-Gln	>100	>50	49.2	26.9

*Mean from three determinations by a stopped-flow, CO_2_ hydrase method^15^. Errors were in the range of 5–10% of the reported values (data not shown).

^a^ Human recombinant isozymes, from ref.^8^^a^.

^b^ Bacterial recombinant enzyme, ref.^14^^b^.

^c^ Protozoan recombinant enzyme, this work.

- Amino acids incorporating a second carboxylic moiety, such as L-Asp, L- and D-Glu, were devoid of activating effects up to concentrations of 50 µM within the assay system, whereas the corresponding compounds with a CONH_2_ moiety, i.e, L-Gln and L-Asn showed modest activating effects, with activation constants in the range of 26.9–32.5 µM.
- Moderate-weak TvaCA1 activation was also observed for the following amino acid derivatives: L-and D-His as well as L- and D-Phe, which showed K_A_‘s ranging between 16.3 and 24.5 µM.
- A number of amino acid and amine derivatives investigated here acted as moderate-effective activators, with K_A_‘s ranging between 8.3 and 14.5 µM. They include: L- and D-DOPA, histamine, dopamine, serotonin, (2-Aminoethyl)pyridine/piperazine and morpholine (**16–18**), the aminomethyl derivative of pyridine **15**, and L-adrenaline **19**.
- The most effective TvaCA1 activators were L-and D-Trp, L-and D-Tyr and 4-amino-Phe **11**, which showed K_A_‘s ranging between 3.0 and 5.1 µM.
- Small structural changes in the activator molecule have important consequences for the activation. For example, in the case of Phe, both the L- and D-enantiomers showed rather modest activating effects. The introduction of *p*-hydroxy moieties on the phenyl ring, as in L-and D-Tyr, led to a marked increase in the activating effects, but the introduction of a second phenolic OH moiety, as in L-and D-DOPA, diminished again the activating properties.
- The activation profile of TvaCA1 with compounds **1–24** is quite different from those of other enzymes, such as hCA I and II or EcoCAβ, but no TvaCA1-selective activators were detected so far.

## 4. Conclusions

We report the first activation study of the β-class CA encoded in the genome of the protozoan pathogen *T. vaginalis*, TvaCA1. In a series of 24 amino acid and amine activators, derivatives incorporating a second carboxylic moiety, such as L-Asp, L- and D-Glu, were devoid of activating effects up to concentrations of 50 µM within the assay system, whereas the corresponding compounds with a CONH_2_ moiety, i.e. L-Gln and L-Asn showed modest activating effects, with activation constants in the range of 26.9 − 32.5 µM. Moderate activation has been observed with L- and D-DOPA, histamine, dopamine, serotonin, (2-Aminoethyl)pyridine/piperazine and morpholine (K_A_’s ranging between 8.3 and 14.5 µM), whereas the best activators were L-and D-Trp, L-and D-Tyr and 4-amino-Phe, which showed K_A_’s ranging between 3.0 and 5.1 µM. Understanding in detail the activation mechanism of β-CAs may be relevant for the design of enzyme activity modulators with potential clinical significance.
